# Supplementary material for: Conditional chemoconnectomics (cCCTomics) as a strategy for efficient and conditional targeting of chemical transmission
Source: eLife. 2024 Apr 30;12:RP91927. doi: 10.7554/eLife.91927 (PMC11060718; doi:10.7554/eLife.91927)
Supplement: Figure 3—source data 3. [file elife-91927-fig3-data3.zip › Figure 3 source data3 part 1.zip!/Figure 3-Source data 3-part1/1071-1-CRISPResso_Report_osfbjvga.zip!/CRISPResso_on_1071_3/1c.Alignment_barplot.pdf]

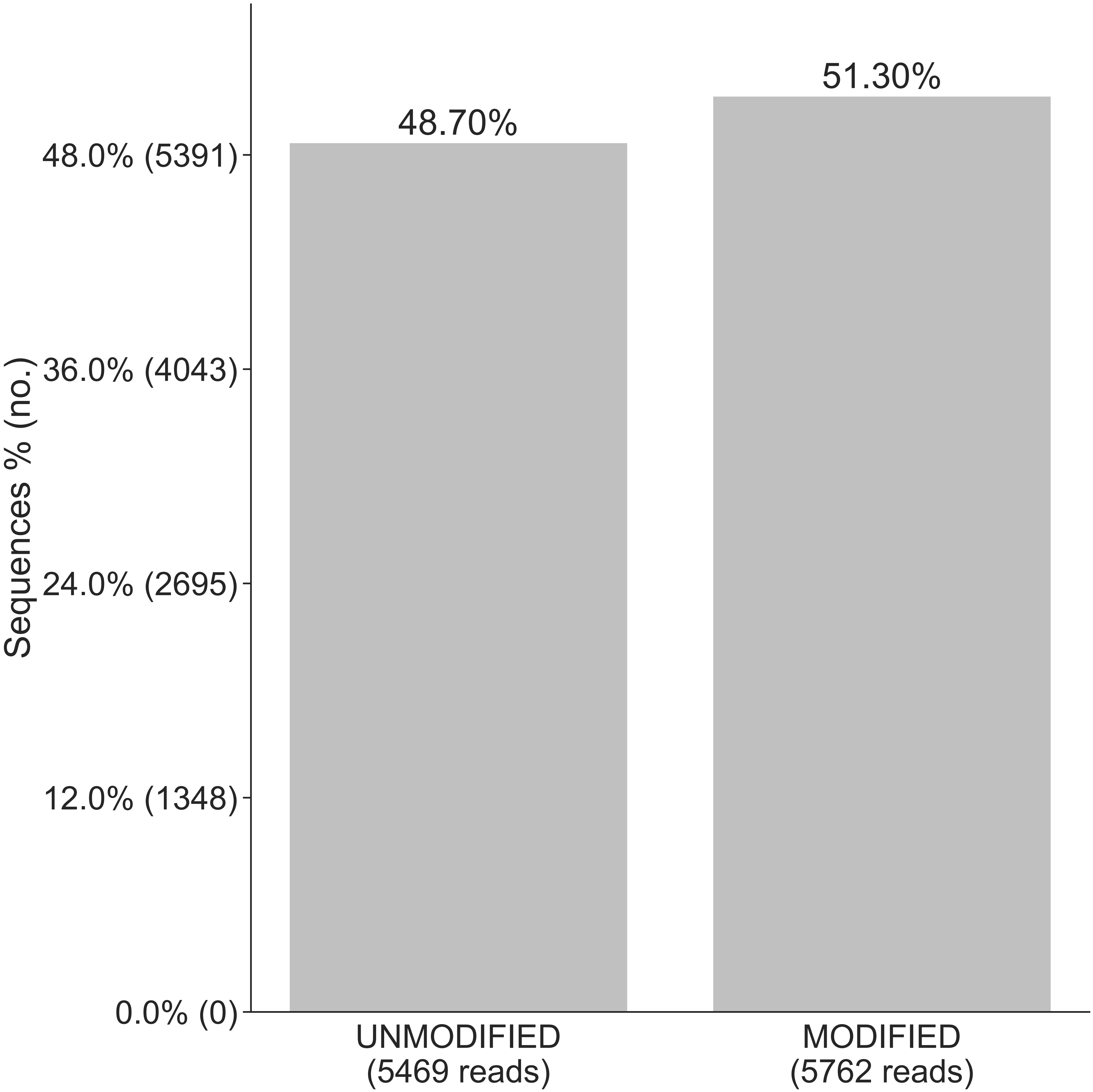

Sequences % (no.)
48.0% (5391)
36.0% (4043)
24.0% (2695)
12.0% (1348)
0.0% (0)
48.70%
51.30%
UNMODIFIED
(5469 reads)
MODIFIED
(5762 reads)
